# Multi Modal Feature Extraction for Classification of Vascular Dementia in Post-Stroke Patients Based on EEG Signal

**DOI:** 10.3390/s23041900

**Published:** 2023-02-08

**Authors:** Sugondo Hadiyoso, Hasballah Zakaria, Paulus Anam Ong, Tati Latifah Erawati Rajab

**Affiliations:** 1 School of Electrical Engineering and Informatics, Institut Teknologi Bandung, Bandung 40116, Indonesia; 2 School of Applied Science, Telkom University, Bandung 40257, Indonesia; 3 Department of Neurology, Dr. Hasan Sadikin General Hospital, Bandung 40161, Indonesia

**Keywords:** vascular dementia, post stroke, quantitative EEG, classification

## Abstract

Dementia is a term that represents a set of symptoms that affect the ability of the brain’s cognitive functions related to memory, thinking, behavior, and language. At worst, dementia is often called a major neurocognitive disorder or senile disease. One of the most common types of dementia after Alzheimer’s is vascular dementia. Vascular dementia is closely related to cerebrovascular disease, one of which is stroke. Post-stroke patients with recurrent onset have the potential to develop dementia. An accurate diagnosis is needed for proper therapy management to ensure the patient’s quality of life and prevent it from worsening. The gold standard diagnostic of vascular dementia is complex, includes psychological tests, complete memory tests, and is evidenced by medical imaging of brain lesions. However, brain imaging methods such as CT-Scan, PET-Scan, and MRI have high costs and cannot be routinely used in a short period. For more than two decades, electroencephalogram signal analysis has been an alternative in assisting the diagnosis of brain diseases associated with cognitive decline. Traditional EEG analysis performs visual observations of signals, including rhythm, power, and spikes. Of course, it requires a clinician expert, time consumption, and high costs. Therefore, a quantitative EEG method for identifying vascular dementia in post-stroke patients is discussed in this study. This study used 19 EEG channels recorded from normal elderly, post-stroke with mild cognitive impairment, and post-stroke with dementia. The QEEG method used for feature extraction includes relative power, coherence, and signal complexity; the evaluation performance of normal-mild cognitive impairment-dementia classification was conducted using Support Vector Machine and K-Nearest Neighbor. The results of the classification simulation showed the highest accuracy of 96% by Gaussian SVM with a sensitivity and specificity of 95.6% and 97.9%, respectively. This study is expected to be an additional criterion in the diagnosis of dementia, especially in post-stroke patients.

## 1. Introduction

Dementia is a common symptom of neurological disorders that represents a decreased cognitive function in the brain [1]. These symptoms include memory loss, thinking, judgment, language, complex motor skills, and other intellectual functions. The most common form of dementia after Alzheimer’s dementia (AD) is vascular dementia (VaD), contributing about 20% in North America and Europe, and about 30% in Asia and developing countries [2]. Vascular dementia is closely related to cerebrovascular disease [3]. Stroke, hypertension, diabetes mellitus, obesity, cholesterol, and heart fibrillation are closely related to vascular dementia [4]. Among these vascular diseases, stroke is most often associated with VaD [5].

Stroke is a significant cause of physical disability and cognitive impairment. However, the decline in cognitive function is often negligible compared to physical disability. Though cognition ability also significantly contributes to patients’ quality of life. Minor strokes can even affect executive and cognitive function, consequently affecting participation, quality of life, and ability to work. Approximately 30% of stroke patients develop dementia within six months of stroke [6]. It is also estimated that another 20–25% will have delayed dementia [7]. Riskesdas data recorded the prevalence of stroke in Indonesia at 7% and increased to 10.9% in 2018 [8]. The proportion of stroke increases at the age of more than 55 years by an amount >30%, and is directly proportional to age. Indonesia, with a very large demographic of the elderly population, coupled with the risk factors of stroke, will provide a new problem, namely dementia, and particularly vascular dementia. Accurate diagnosis is needed to inhibit disease progression and determine appropriate therapeutic management to maintain the quality of life of post-stroke patients.

The VaD diagnostic process is quite complex, starting with the patient’s or family’s complaints, and then having to go through the stages of clinical diagnosis of cognitive impairment that is severe enough to meet the criteria for dementia. It must be proven that dementia results from cerebrovascular disease, including stroke, as evidenced by brain imaging. According to the Vascular Impairment of Cognition Classification Consensus Study (VICCCS) [9], mild vascular cognitive impairment is established when there is a disturbance in at least one or more cognitive domains (executive function, attention, and memory, in addition to language and visuospatial functions). However, they do not interfere with basic daily activities or mild disturbances in complex/instrumental activities. Meanwhile, VaD is enforced when a deficit in one or more cognitive domains is severe enough to interfere with basic and instrumental daily activities [10].

Screening tests for neurocognitive disorders that can be used are the Mini-Mental State Examination (MMSE) or the Montreal Cognitive Assessment (MoCA) [11]. Furthermore, brain imaging techniques, including MRI, CT-Scan, and PET-Scan, are essential diagnostic tools in post-stroke dementia [6]. However, brain imaging is expensive and is not recommended for routine examinations in the short term [12]. An electroencephalogram (EEG) can be a potential tool for observing decreased brain function. An EEG can be essential for studying cortical brain abnormalities associated with cognitive decline and dementia [13]. An EEG is a low-cost, non-invasive method that has the sensitivity to detect early dementia and even measure its severity [14]. Quantitative EEG (QEEG) in cases of Alzheimer’s dementia has been reported in [15,16,17]. Characterization of EEG signals in cases of Lewy Body dementia reported in [18,19].

To the best of our knowledge, EEG studies on cases of vascular dementia, especially in post-stroke patients, are still few. The EEG characterization of VaD was reported by Sheng et al. by measuring signal strength using the S-transformation. EEG theta waves in VaD patients have high energy [20]. Another study carried out the characterization and detection of VaD, in which VaD patients resulted in smaller signal complexity values and alpha/theta ratios than normal [21]. Recently, Musa et al. classified patients with VaD and healthy subjects by extracting energy using the Hilbert Huang Transform (HHT). This feature was then classified using an extreme learning machine and obtained 94.4% accuracy [22]. However, previous studies have not carried out the characterization and classification of early-stage vascular dementia. In particular, the research subject in Indonesia.

This study proposed an EEG wave characterization method to classify VaD in post-stroke patients with mild cognitive impairment and dementia by calculating and analyzing QEEG parameters. The proposed feature extraction method is spectral analysis, coherence, and signal complexity. The result of this calculation becomes a feature vector to be validated using machine learning. Simulations were conducted to classify normal elderly, post-stroke patients with mild cognitive impairment, and post-stroke dementia. Support Vector Machine (SVM) and k-Nearest Neighbor (k-NN) are used to evaluate the performance of the proposed feature extraction method. Combining these characterization methods is expected to provide a complete description of the analysis to increase detection accuracy and, ultimately, become a reliable additional diagnostic instrument.

## 2. Materials and Methods

### 2.1. Classification Design

**Figure 1:**
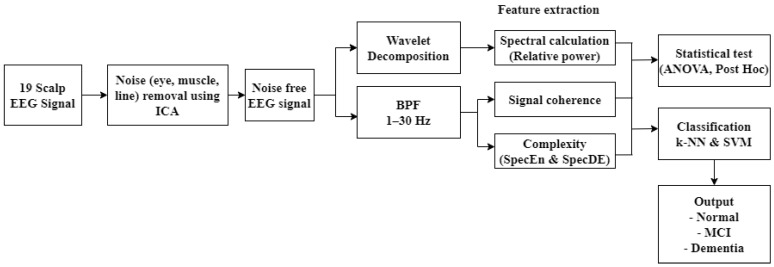
Classification design.

The classification scheme of vascular dementia in post-stroke patients using EEG signal analysis is presented in Figure 1. In the first stage, nineteen scalp EEG signals were denoised using independent component analysis (ICA). Wavelet transform was then used for the segmentation of EEG bands. The next stage was feature extraction by calculating spectral power, coherence, and complexity. These features were further referred to as predictors in the classification of normal, post-stroke mild cognitive impairment (MCI), and post-stroke dementia using k-nearest neighbor (K-NN) and support vector machine (SVM). Performance evaluation values included accuracy, sensitivity, specificity, precision, and F1-score.

### 2.2. Subject Criteria and EEG Recording (Primary Datasets)

**Figure 2:**
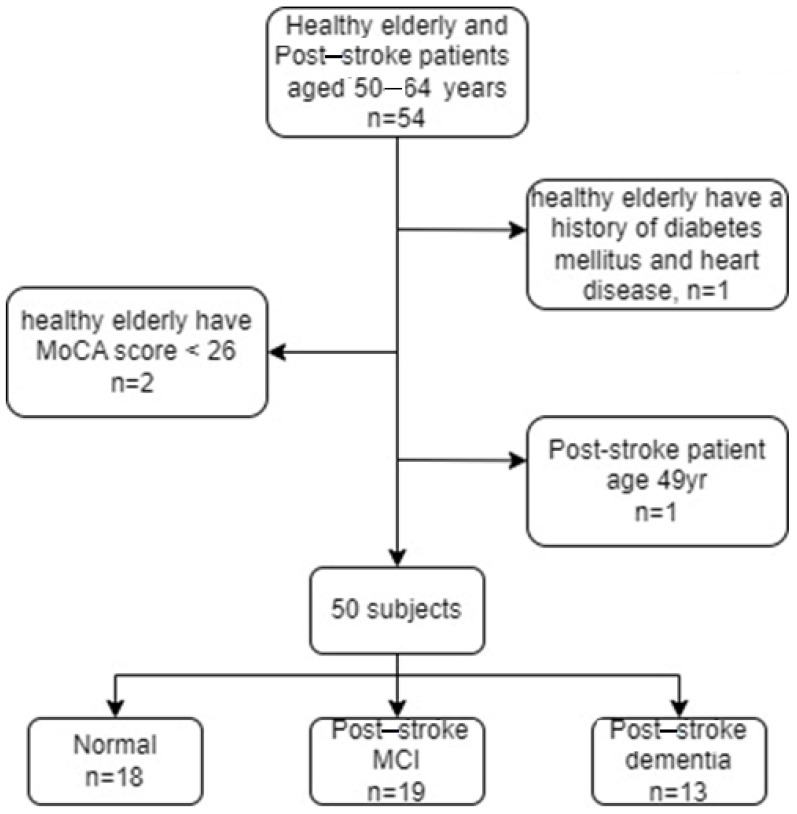
Summary of subject selection criteria.

This study ran from November 2019 to April 2022. The recruitment and data collection of subjects were carried out at the neurological clinic and diagnostic center of Hasan Sadikin General Hospital, Bandung. The subject criteria used in this study were based on the recommendations of a neurologist (neurobehavior consultant) and the Indonesian Neurologist Association (PERDOSSI) after clinical examination, neuropsychology, and brain imaging were carried out. The inclusion criteria for patients included stroke after three months, with a lacunar or subcortical infarct, age 50–64 years, and minimum education in junior high school or equivalent. The MoCA-Indonesia (INA) score is less than 19, and has impaired basic and instrumental activities of daily living for post-stroke patients with dementia. Meanwhile, for patients with mild vascular cognition, if the MoCA-INA score is between 19–25, there are no disturbances in basic daily activities or mild disturbances in daily instrumental activities. Figure 2 presents a summary of the subject selection criteria.

The normal control inclusion criteria included an age between 50–64 years, a minimum junior high school education, an MoCA-INA score ≥ 26, and ability to read and write. Neurological physical examination results did not find focal neurological deficits on neurological clinical examination by a neurologist. Exclusion criteria for both sample groups were subjects with aphasia and no sensory disturbances in hearing, vision, movement disorders, and a history of cerebral diseases, such as epilepsy, severe head injury, multiple sclerosis, brain tumor, history of brain surgery, and alcoholism, determined by a neurologist. The total number of participants was 50 subjects, consisting of 18 subjects with normal categories, 19 post-ischemic stroke patients with MCI, and 13 post-ischemic stroke patients with dementia. All subjects involved in this study were asked to fill out an informed consent form. Clinical data from each group are presented in Table 1.

**Figure 3:**
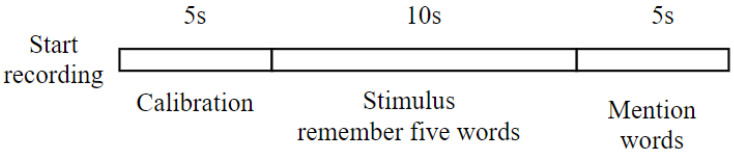
EEG recording of memory, starting with calibration to ensure the condition of the electrodes and then continuing with the stimulus.

The next step was recording the EEG signal using the Cadwell EasyIII clinical standard EEG device. EEG was recorded on 19 channels, including Fp1, Fp2, F7, F3, Fz, F4, F8, T3, C3, Cz, C4, T4, T5, P3, Pz, P4, T6, O1, and O2 with electrode placement following the 10–20 international system. The signal was recorded with a sampling frequency of 250 Hz, a sensitivity of 0.5 µV, and an ADC resolution of 18 bits. Line noise with a frequency of 50–60 Hz was removed using an analog front end with a power of >110 dB. EEG recording was carried out under several conditions, namely relaxed with eyes closed, relaxed with eyes open, given a photic stimulus, and undertaking cognitive tests, including memory. However, the focus of signal processing was on the memory state. In the memory recording, subjects were given verbal instructions to memorize five words and were then asked to recall the words they remembered. The design of the EEG recordings during the memory work referred to previous studies [22,23]. Figure 3 shows the EEG recording design. Signal processing was carried out in the phase when the stimulus was given, and the subject mentioned the words.

### 2.3. Alzheimer’s Dataset (Normal vs. MCI)

In this study, signal characterization was also carried out in the Alzheimer’s case dataset, which consisted of EEG recordings from normal elderly subjects and elderly with MCI. This dataset was sourced from research at the Sina and Nour Hospital, Isfahan, Iran. The dataset was collected from 11 healthy elderly subjects and 16 elderly subjects with MCI [17]. All subjects were over 60 years old and had at least a basic education. A psychiatrist examined all of the subjects, including the mini-mental state examination (MMSE), for validation of MCI or normal. Subjects with an MMSE score of more than 26 were normal controls, while subjects with a score of 21–26 were MCI. The Neuropsychiatric Unit Cognitive Assessment Tool (NUCOG) was also used to confirm MCI. 

The EEG signal was recorded using Galileo NT EEG, EBneuro. Resting EEG recordings with eyes closed were performed for 30 minutes. Nineteen channels were recorded, including Fp1, Fp2, F7, F3, Fz, F4, F8, T3, C3, Cz, C4, T4, T5, T5, P3, Pz, P4, T6, O1, and O2, with a sampling frequency of 256 Hz.

### 2.4. Pre-Processing the EEG Signal

**Figure 4:**
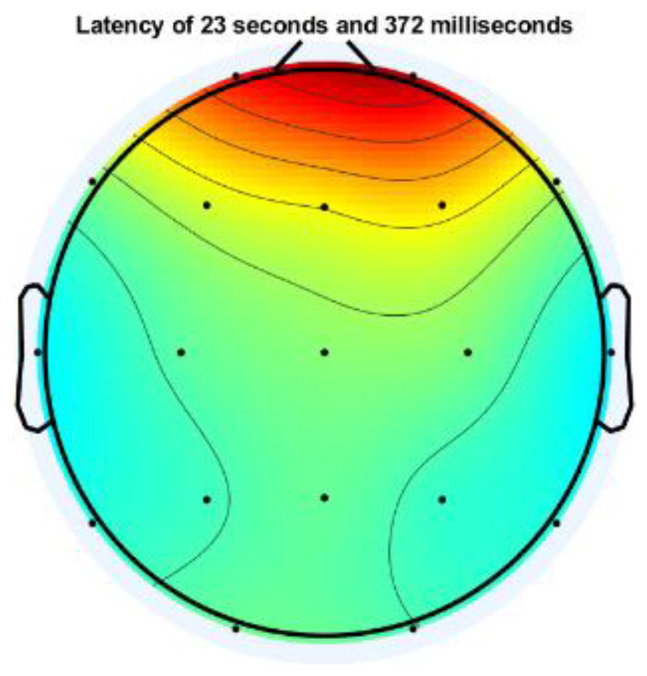
Topographic plot of one EEG channel contaminated with eye artifact.

Signal pre-processing was performed on the raw EEG signal to remove eye artifact noise, baseline wandering, and line and muscle noise. Signal pre-processing is one of the critical issues in preparing the EEG signal for the next processing stage, where the EEG signal is free from noise. Low-frequency and high-frequency noise commonly contaminate EEG signals, even with high power. Line and muscle noise comes with a high frequency, while eye noise has a low frequency. At this stage, two approaches are used to eliminate the noise: Independent Component Analysis (ICA) and a digital BPF filter at a cut-off frequency of 1–30 Hz. The ICA process is carried out using the EEGLAB toolbox in MATLAB. The topographic plot of the channel containing noise representing non-cortical activity (eyeball and/or muscle movement potential) is shown in Figure 4.

**Figure 5:**
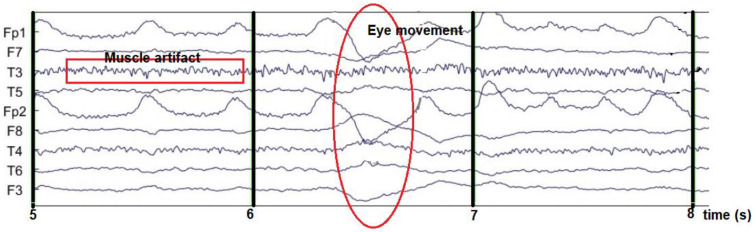
EEG signal contaminated with noise.

Meanwhile, Figure 5 shows the EEG signal mixed with eye artifacts and muscle noise. The results of the ICA decomposition can then be visually observed for noise-containing EEG channels. The noise source is then removed with the EEGLAB tool.

In the coherence and signal complexity calculation phase, previously, the signal was filtered with a range of 1–30 Hz. This step aims to obtain the fundamental frequency from delta to beta of the EEG signal. A high pass filter with a cut-off frequency of 1 Hz and a low pass filter with a cut-off of 30 Hz is applied at this stage. Both high and low pass filters are designed using Butterworth, with a passband ripple of 1 dB and a stopband attenuation of 80 dB.

### 2.5. Feature Extraction

In this study, feature extraction computes essential information to differentiate normal EEG, MCI, and dementia. The proposed feature extraction methods include spectral analysis, coherence, and complexity. The results at this stage are used as a predictor in the classification scheme.

#### 2.5.1. Spectral Analysis

Spectral analysis is one of the most common methods used in EEG signal quantification. This analysis measures the power spectral density (power spectrum), which reflects the power distribution of a signal over frequency. Furthermore, in this study, the power spectral density was estimated using the Welch method with a window of 2 s and an overlap of 75%. Power spectral estimation using Welch, calculated in each EEG band, is expressed by Equation (1) below.

(1) $${\overset{ˇ}{P}}_{xx}^{w}=\frac{1}{U}\overset{U-1}{\underset{i=0}{\sum }}{\overset{ˇ}{P}}_{xx}^{i}\left(f\right)$$

where ${\overset{ˇ}{P}}_{xx}^{i}\left(f\right)$ = spectral estimation $X_{i}\left(n\right)$

${\overset{ˇ}{P}}_{xx}^{w}$ = spectral Welch

*U* = window function

**Figure 6:**
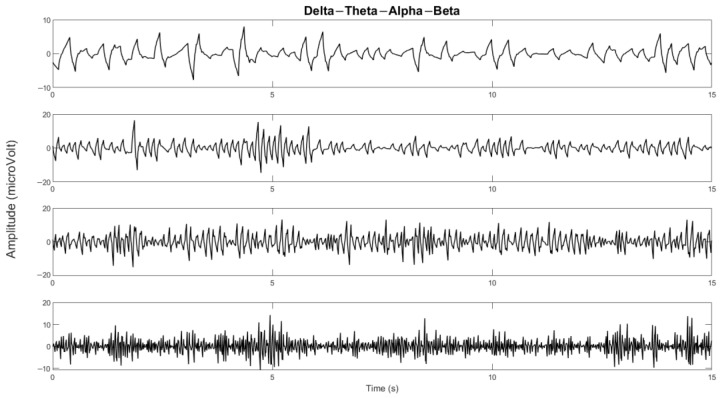
The delta, theta, alpha, and beta bands of one of the channels.

The delta, theta, alpha, beta, and gamma bands were segmented using wavelet decomposition with Daubechies-2 (DB2) as the basis or mother wavelet. The Daubechies family was chosen for its good performance, as reported in [24,25]. In more detail, DB2 in EEG cases has been commonly used and shows good performance, as reported in [26,27]. The signal is decomposed into five levels for segmenting these bands with a resampling frequency of 240 Hz. The wavelet decomposition and corresponding EEG frequency bands are presented in Table 2. The segmented signal according to the frequency band is presented in Figure 6.

Welch estimates the absolute power that depends on the amplitude value of each individual. So, it gives very varied results. Therefore, it is necessary to normalize the absolute value, called relative power. The relative power is the ratio between the absolute power of the frequency bands to each other, written in Equation (2) below.

(2) $$P_{relative}=\frac{P_{abs(i)}}{\sum_{fL}^{fH}P_{abs}}$$

where $P_{abs(i)}$ is determined by the selected frequency band and [*fL*, *fH*] are the delta, theta, alpha, beta, and gamma bands.

#### 2.5.2. EEG Signal Coherence

**Figure 7:**
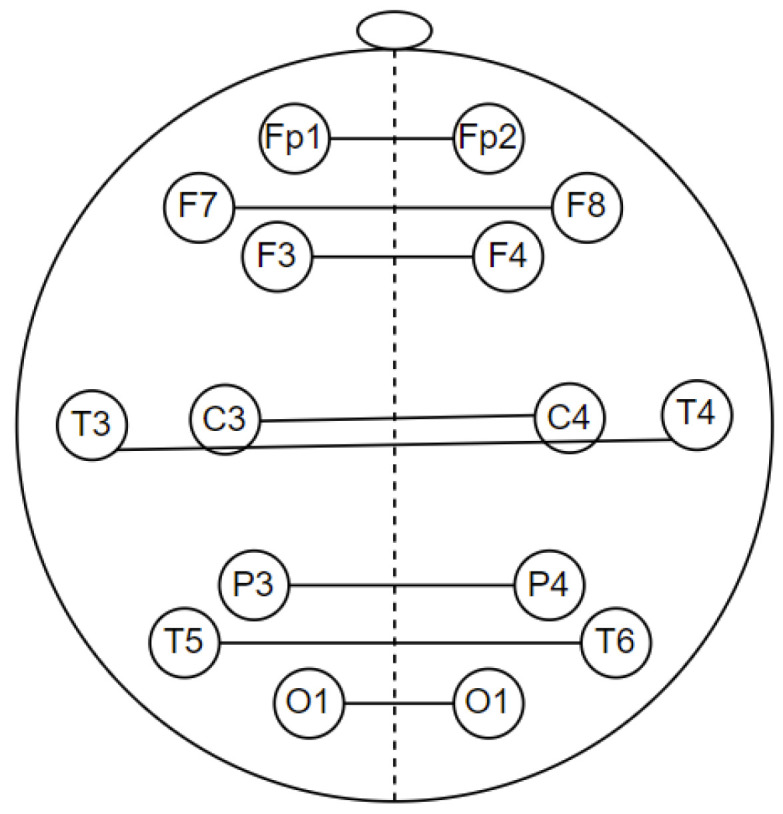
Electrode pairs for interhemisphere coherence calculations.

EEG signal coherence analysis was performed to observe the functional connectivity of the brain [29]. In quantitative EEG, coherence is commonly used to measure functional connectivity in the human cortex [30]. Coherence is a measure of synchronization between two signals mainly based on phase consistency. In this study, coherence was calculated for intrahemisphere and interhemisphere pairs with a frequency range of 1–30 Hz. Intrahemispheric coherence was calculated at the electrodes in one hemisphere area. It consists of the right intrahemisphere and the left intrahemisphere. Meanwhile, interhemispheric coherence was calculated at electrodes in different hemisphere areas, as shown in Figure 7. Both intrahemispheric and interhemisphere electrode pairs are presented in Table 3.

Coherence is a measure of synchronization between two signals mainly based on phase consistency. A high coherence value occurs when the phase difference between channels tends to be constant. Coherence can be expressed by dividing the square of the cross-spectral density of the two channels by the product of the power spectral density of the two channels. Coherence ($C_{ab}$) from signals a and b calculated using the power spectral density ($P_{aa}$ dan $P_{bb}$) and cross-power spectral density ($P_{ab}$); Equation (3) shows the calculation of coherence [31].

(3) $$C_{ab}\left(f\right)=\frac{{\left|P_{ab}\left(f\right)\right|}^{2}}{P_{aa}\left(f\right)*P_{bb}\left(f\right)}$$

where *f* is frequency.

#### 2.5.3. Signal Complexity Measurement

The feature extraction of the EEG signal at this stage is carried out with a complexity approach to calculate the degree of signal irregularity/randomness. The complexity approach in this research is based on entropy theory. The complexity of the EEG signal is estimated using spectral entropy and a new method called spectral dispersion entropy. These methods are described in the following sub-section.

##### Spectral Entropy

Spectral entropy estimates the randomness of the signal based on the spectral amplitude over a specified frequency range [32]. Spectral entropy is calculated using the Shannon entropy formula, which is applied to the power spectral density of the EEG signal using Equation (7) [33]. A high spectral entropy value represents a high level of signal complexity.

(4) $$SpecEn=-\overset{fh}{\underset{fi=0}{\sum }}Pflog_{2}\left(Pf\right)$$

with *Pf* is *power spectral density* of the specified frequency band, while *fi* and *fh* is the limit frequency of the signal.

##### Dispersion Entropy

Recently, dispersion entropy (DisEn) has received significant attention, where DisEn has been shown to outperform sample entropy and permutation entropy. DisEn was first proposed by Azami in 2016 [34,35]. Dispersion entropy converts the data into a new signal with several predetermined patterns, and then the probability of the occurrence of the pattern is calculated. The DisEn calculation method is based on a new signal pattern mapping function with the following parameters: length *m* template; the number of classes *c* represents the number of patterns, and the time delay *d*.

The DispEn algorithm includes four main steps for univariate signal $N:x=\left\{x1,x2,\ldots ,xN\right\}$:

1. Take a number of linear and nonlinear approaches to mapping $xj\left(j=1,2,\ldots ,N\right)$ to class *c* from 1 to *c*. The normal cumulative distribution function (NCDF) is used to map x to $y\left(y=y1,y2,\ldots ,yN\right)$ from 0 to 1. The signal has *m* members, and each member is an integer from 1 to *c*.
2. The number of possible dispersion patterns for each time series is defined as $z_{i}^{m,c}$= $c^{m}$. Each embedding vector $z_{i}^{m,c}$ has dimensions with length m template, time delay d, and number of class c, which represents the number of patterns. $z_{i}^{m,c}=\left\{z_{i}^{c},z_{i+d}^{c},\ldots z_{i+\left(m-1\right)d}^{c}\right\}$, i=1,2,…,N−(m−1)d create embedding vector $z_{i}^{m,c}$ mapped to a dispersion pattern $\pi_{v_{0}v_{1}\ldots v_{m-1}}$, where $z_{i}^{c}=v_{0},z_{i+d}^{c}=v_{1},\ldots ,z_{i+\left(m-1\right)d}^{c}=v_{m-1}.$
3. The number of dispersion pattern $\pi_{v_{0}v_{1}\ldots v_{m-1}}$ represented as $p\left(\pi_{v_{0}v_{1}\ldots v_{m-1}}\right)$ for $z_{i}^{m,c}$. For the calculation of the frequency of occurrence of $c^{m}$, Equation (5) is used.
  (5) $$p\left(\pi_{v_{0}v_{1}\ldots v_{m-1}}\right)=\frac{Number\left\{i|i\leq N-\left(m-1\right)d,z_{i}^{m,c}has\;\;type\;\;\pi_{v_{0}v_{1}\ldots v_{m-1}}\right\}}{N-\left(m-1\right)d}$$
4. Based on the probability of occurrence of the dispersion pattern, DispEn is calculated using the following mathematical expression.
  (6) $$DispEn\left(signal,m,c,d\right)=-\sum_{\pi =1}^{c^{m}}p\left(\pi_{v_{0}v_{1}\ldots v_{m-1}}\right)\cdot ln\left(p\left(\pi_{v_{0}v_{1}\ldots v_{m-1}}\right)\right)$$

##### Spectral Dispersion Entropy

Spectral dispersion entropy is an extension of spectral entropy where the spectral amplitude of the signal is calculated using dispersion entropy. Previously, the probability of the appearance of the amplitude in the direct power spectral was calculated using Shannon’s theory. In spectral dispersion entropy, the power spectral randomness level is calculated by estimating the similarity of the dispersion pattern from a number of spectral series. Dispersion entropy is calculated with length *m* template = 2; the number of classes *c* = 6, which represents the number of patterns, and the time delay *d* = 1.

### 2.6. Significant Test and Performa Evaluation

In testing the significance of the difference between normal, post-stroke MCI, and post-stroke dementia, post hoc multiple comparison with analysis of variance (ANOVA) was used. In this study, the pair test of the two groups had a significant difference if the *p*-value < 0.05.

**Figure 8:**
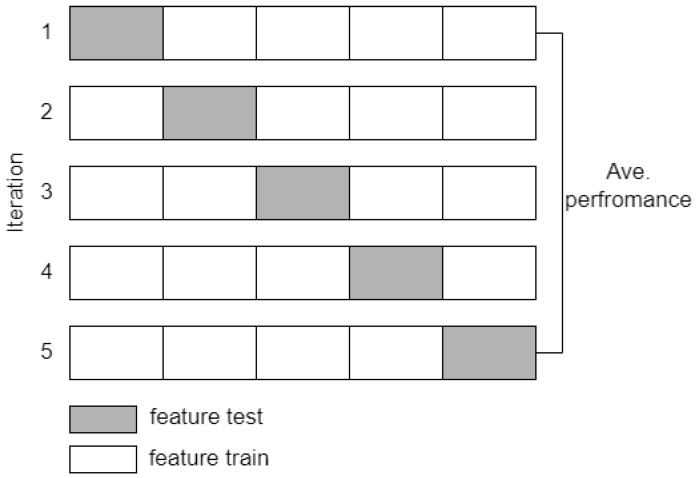
Cross validation scheme with k = 5.

The feature extraction method proposed in this study was also evaluated by classification simulation using the SVM and k-NN algorithms. The goal is to obtain the accuracy value as an additional analysis of the significance test. The calculated EEG features, including spectral power, coherence, and complexity, are then referred to as predictors in the stage classification. The cross-validation method divides the training and test features with k = 5 iterations, as illustrated in Figure 8. The final accuracy value is the average result of each classification iteration. The performance parameters of the proposed method are accuracy, sensitivity, and specificity, which are calculated using Equations (7)–(9) [36]. Other performance parameters that are measured to confirm accuracy are precision and F1-score. Mathematically, precision and F1-score are expressed in Equations (10) and (11) [36].

(7) $$Accuracy=\frac{True\;positive+True\;negative}{True\;positive+False\;positive+True\;negative+False\;negative}$$

(8) $$Sensitivity=\frac{True\;positive}{True\;positive+False\;negative}$$

(9) $$Specificity=\frac{True\;negative}{True\;negative+False\;positive}$$

(10) $$Precision=\frac{True\;positive}{True\;positive+False\;positive}$$

(11) $$F1-score=2\times \frac{Precision\;\times \;Sensitivity}{Precision+Sensitivity}$$

## 3. Results

This section describes the study results related to the feature extraction results from each method. The results are presented in graphs and tables, followed by relevant clinical explanations. This chapter also presents the results of the validation of the proposed method in the form of classification accuracy.

### 3.1. Power Spectral Characteristics on the Primary Dataset

**Figure 9:**
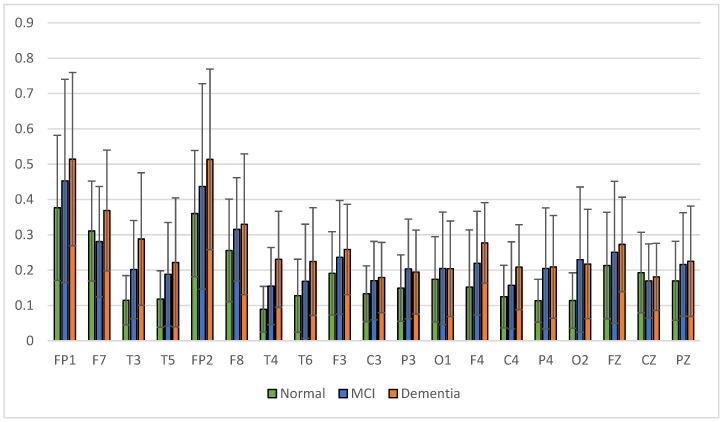
Relative power delta band.

**Figure 10:**
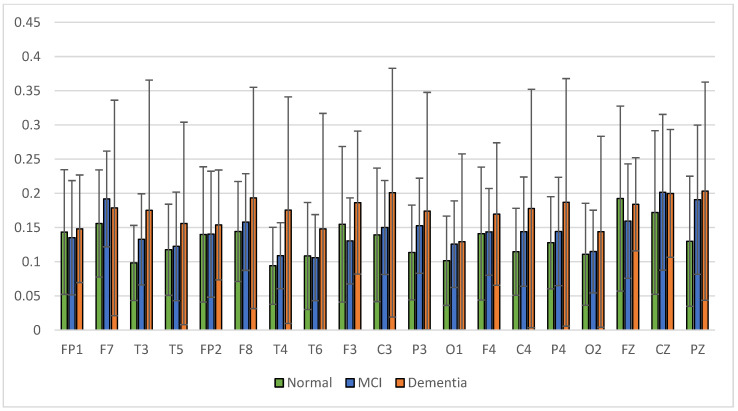
Relative power theta band.

**Figure 11:**
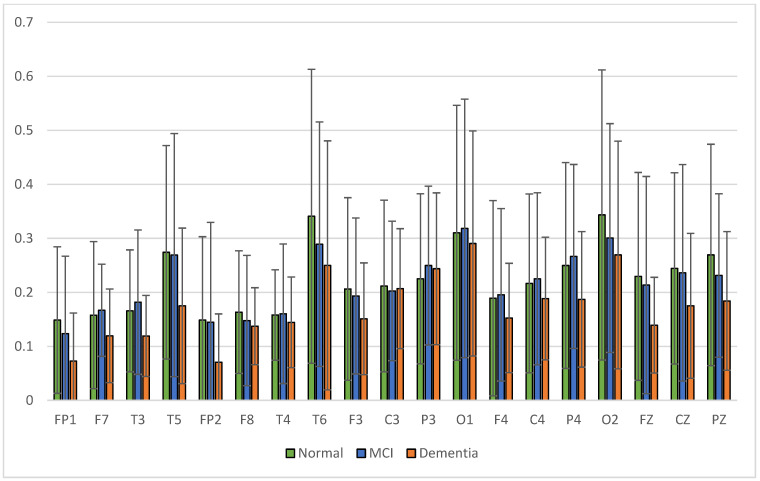
Relative power alpha band.

**Figure 12:**
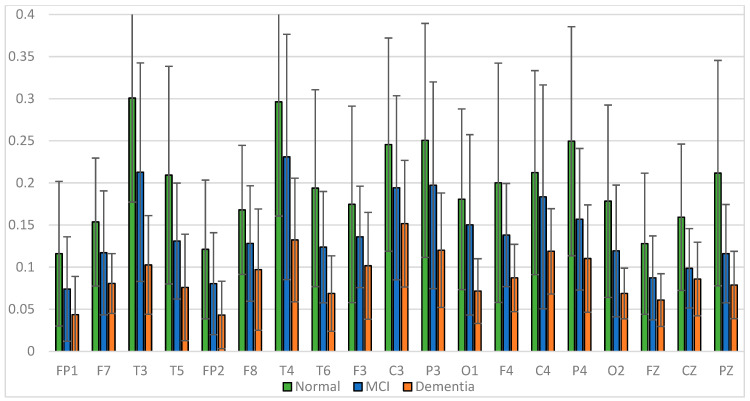
Relative power beta band.

The results of relative power measurements on 19 EEG channels for each group are shown in Figure 9, Figure 10, Figure 11 and Figure 12.

The average relative power of each group showed significance in the delta rhythm, where post-stroke dementia and MCI groups tended to be higher than the normal group. While it was significantly higher in the beta rhythm, the normal group was higher than the post-stroke MCI and dementia. Decreased strength of beta rhythms in MCI and dementia is associated with reduced focus or concentration on working memory tasks. The power of the delta and beta rhythms showed a correlation with the severity of dementia, where patients with dementia had the highest delta power and the lowest beta power. The significance of the difference with *p* < 0.05 is shown in Table 4 below.

### 3.2. Power Spectral Characteristics of the Alzheimer’s Dataset

**Figure 13:**
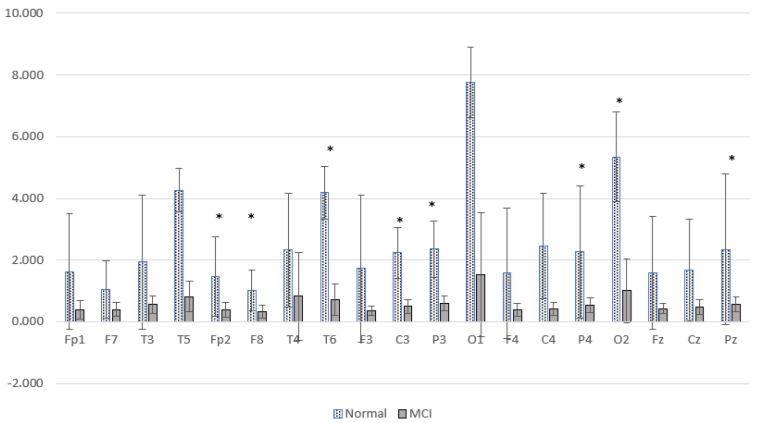
Comparison of the relative power of high and low frequency MCI and normal groups. * *p* < 0.05.

Power spectral characterization of the Alzheimer’s dataset has been reported in a previous study [37], represented by a comparison of the relative power of high and low frequencies. Relative power alpha-beta (RAP + RBP) is a high-frequency representation, and relative power delta-theta (RDP + RTP) is a low-frequency representation. Figure 13 presents a comparison of the low relative power of the MCI and normal groups.

Figure 13 shows the difference between the two brain conditions; the relative power at high frequencies of normal subjects is higher than that of MCI subjects. Significant differences were found at Fp2, F8, T6, C3, P3, P4, Pz, and O2. The increase in delta power and decrease in alpha power were spread over all observed brain cortical areas. In general, these results are similar to cases of post-stroke cognitive impairment. There was a characteristic change in EEG activity marked by shifting the power signal to a lower frequency.

### 3.3. Coherence Characteristics on the Primary Dataset

**Figure 14:**
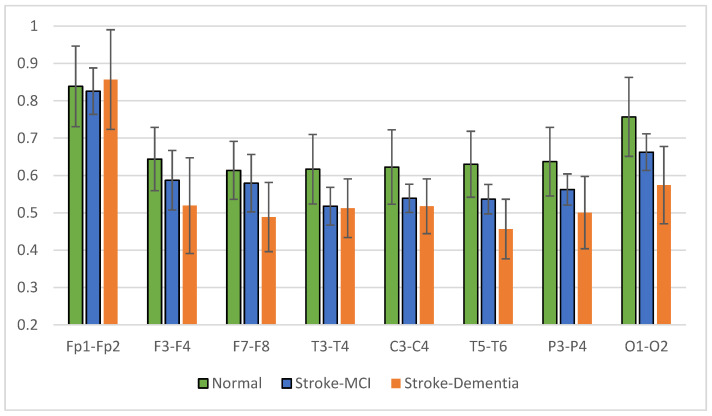
Interhemispheric coherence value of each electrode pair.

The signal coherence of the eight and twenty-eight electrode pairs, as shown in Table 3, is calculated using Equation (3). Interhemispheric coherence calculates the EEG coherence of the right and left hemispheres for inline electrodes. The results of the average interhemispheric coherence for each electrode pair are presented in Figure 14. The results of interhemispheric coherence show that, in general, the mean coherence in post-stroke patients with cognitive impairment tends to be lower than the normal group for all electrode pairs. Significant differences (*p* < 0.05) were found in the frontal, central, and temporal regions, pairs F7-F8, T3-T4, T5-T6, and P3-P4, as shown in Table 5. While in the results of the post hoc multiple comparison tests, the T5-T6 pairs showed differences between the three groups. Decreased coherence could be expected due to decreased connectivity electricity connecting brain areas.

**Figure 15:**
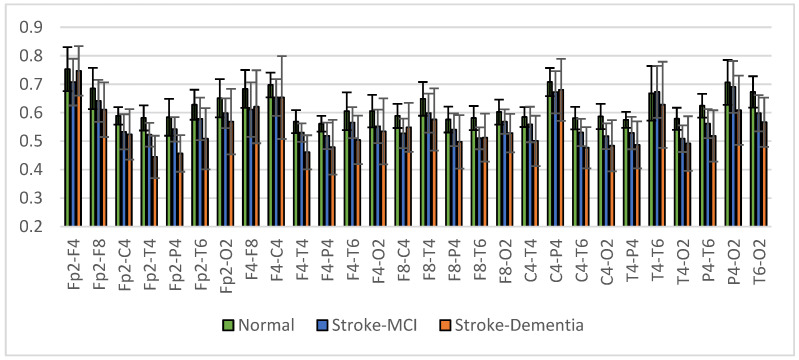
Right intrahemispheric coherence value of each electrode pair.

**Figure 16:**
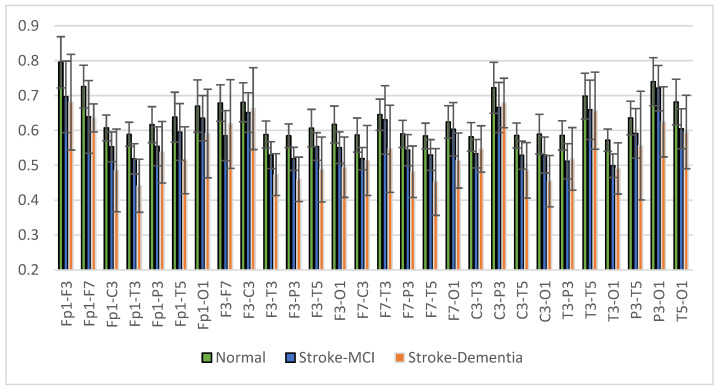
Left intrahemispheric coherence value of each electrode pair.

The mean right intrahemispheric coherence for each pair of electrodes is presented in Figure 15. The results showed a decrease in right intrahemispheric coherence in patients with cognitive impairment. The pair of electrodes resulted in a *p*-value < 0.05, as shown in Table 6. Meanwhile, the left intrahemispheric mean showed similar characteristics, where people with dementia experienced a decrease in coherence values. Figure 16 depicts the mean left intrahemispheric coherence for each electrode pair. Significant differences with *p* < 0.05 are shown in Table 7.

The average measurement results show that the coherence of the post-stroke patient group with cognitive impairment is generally lower than the normal group. This condition occurs in almost all interhemispheric and intrahemispheric electrode pairs.

### 3.4. Coherence Characteristics on the Alzheimer’s Dataset

Coherence measurements in the Alzheimer’s dataset have been reported in a previous study [38]. The coherence calculations results show that the MCI group’s coherence is lower than the normal elderly subjects. In interhemispheric coherence, significant differences were found in FP1-FP2. Meanwhile, significant differences in intrahemispheric pairs were found in FP2-T4, FP2-F4, FP1-F7, FP1-F3, FP1-P3, FP1-C3, FP1-T3, FP1-T5, F3-O1, FP1-O1, and T3-T5. Coherence measurements in the Alzheimer’s dataset also show differences between normal and pathology. The coherence method can be an attractive feature for normal and pathological classification.

### 3.5. Complexity Characteristics on the Primary Dataset

The signal complexity analysis method is expected to provide differentiating characteristics between the observed groups so that quantitative EEG analysis can be used as a supporting criterion for early diagnosis of post-stroke vascular dementia. The signal complexity calculation method in this study is entropy-based. This calculation is performed on a time series function signal using spectral entropy (SpecEn) and a new method called spectral dispersion entropy (SpecDE).

**Figure 17:**
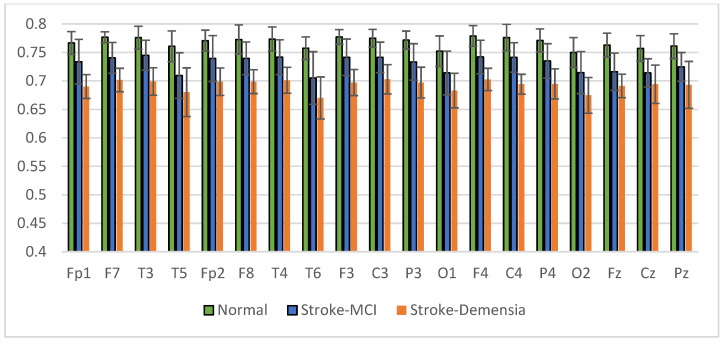
Mean of the SpecEn values of the normal, post-stroke MCI, and dementia.

**Figure 18:**
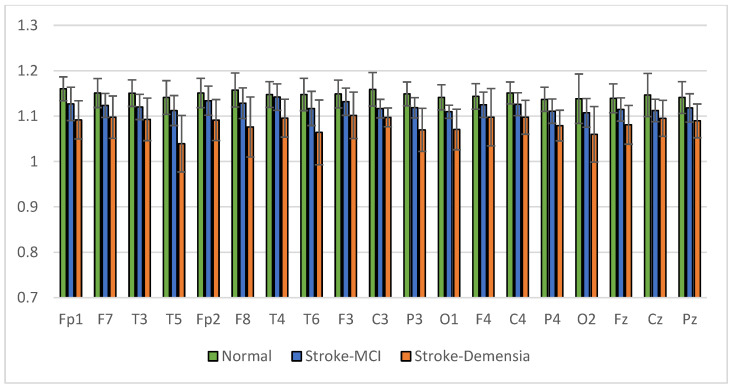
Mean of the SpecDE values of the normal, post-stroke MCI, and dementia.

The average results of SpecEn and SpecDE measurements on 19 electrodes for each group are presented in Figure 17 and Figure 18. The measurement results show that the group with cognitive impairment tends to have a lower signal complexity than the normal group.

### 3.6. Complexity Characteristics on the Alzheimer’s Dataset

**Figure 19:**
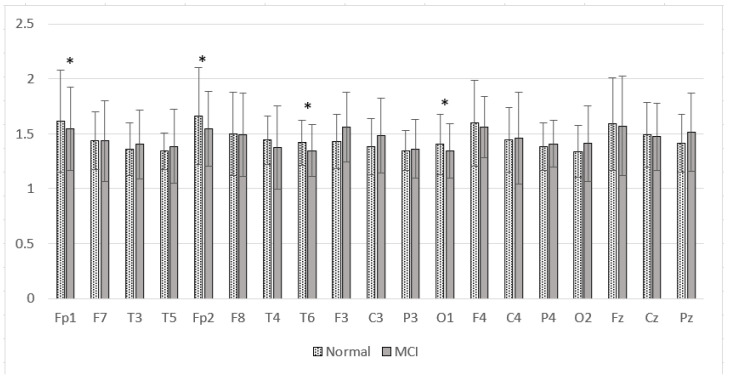
Mean of the SpecEn values of the normal and MCI groups. * *p* < 0.05.

The results of the SpecEn calculations in the Alzheimer’s dataset are presented in Figure 19. Figure 19 shows that the SpecEn values in the MCI group generally tend to be lower than the normal group. Significant differences were found in Fp1, Fp2, T6, and O1. These results indicate a decrease in EEG signal complexity in MCI patients. These characteristics are similar to post-stroke patients with cognitive impairment. From these results, it is hoped that the degree of complexity can be a reliable feature for discrimination between normal subjects and patients with cognitive impairment.

### 3.7. Performance Comparison of SpecEn and SpecDE

The results of the different tests on SpecEn and SpecDE are presented in Table 8. The difference test with *p*-value < 0.05 showed a significant difference between groups.

Based on the significance test, the degree of signal complexity based on SpecEn and SpecDE showed a significant difference between the normal and post-stroke cognitive impairment groups. Significant differences with *p*-value < 0.05 were found across channels for SpecEn and SpecDE. The average complexity value also indicates a relationship between the decrease in signal complexity and the severity of dementia. Therefore, multiple comparison post hoc testing is needed to test the significance between groups, specifically the normal vs. post-stroke MCI and post-stroke MCI vs. post-stroke dementia groups.

Tukey’s post hoc t-test was used for the multiple comparison tests in this study. The test results are presented in Table 9 and Table 10. From this test, it was known that SpecDE analysis provides discriminatory significance for the case of three groups superior to SpecEn. Significant differences for the three groups, with *p* < 0.05, were more in SpecDE than in SpecEn. The post hoc multiple comparison test results for SpecDE showed significant differences between groups at the Fp1, P3, O1, C4, and P4 electrodes. These results will significantly affect the accuracy at the classification stage.

### 3.8. Classification of Normal, Post-Stroke MCI, and Post-Stroke Dementia

In the previous section, the characterization of the EEG signal was discussed in both the primary dataset and the Alzheimer’s dataset. Power spectral, coherence, and complexity analysis methods can produce discriminatory features between classes based on the tests carried out. The main objective of this study is to detect early-stage cognitive impairment in post-stroke patients using the proposed method. The feature extraction result from each method presented in the previous sub-section becomes a feature vector or predictor in the classification stage. The proposed methods are evaluated using automatic classification algorithms, including k-NN and SVM. This test was carried out with several scenarios, as presented in Table 11. Scenarios A, B, C, and D were used to evaluate the performance of each feature extraction method. Meanwhile, the combination of predictors in scenario E was chosen by considering the significance test results.

Several SVM kernels and k-NN types are also used to obtain the highest accuracy. SVM kernels include linear, quadratic, cubic, and gaussian. The penalty parameter used is equal to 1 for all kernels. Specifically, for the gaussian kernel, the parameter scale is set to sqrt (number of predictors). Meanwhile, k-NN includes fine, medium, and cubic k-NN with Euclidean and cubic distance metrics. The number of neighbors for the fine, medium, and cubic k-NN are 1, 10, and 10, respectively. The results of the evaluation of system performance in the EEG classification of normal, post-stroke MCI, and post-stroke dementia for all test scenarios are presented in Table 12.

Table 12 shows that the highest accuracy was 96%, with a specificity and a sensitivity of 95.6% and 97.9%, respectively. The highest accuracy is achieved by scenario E using Gaussian SVM, where coherence features and SpecDE are used as predictors. Combining these features results in higher accuracy than using a single-feature extraction method. Compared to other characterization methods, the most dominant coherence feature contributes to high accuracy. It can be seen in the scenario B simulation that the coherence feature provides an accuracy of up to 94%. Another concerning finding is that the proposed spectral dispersion entropy method produces a higher classification accuracy than the spectral entropy for all classification methods. SpecDE can produce up to 80% accuracy. From this simulation, it can be concluded that SpecDE provides better discrimination features than spectral entropy, as seen in the significance test results presented in the previous subsection.

The proposed method was also tested using the ten-cross validation technique. The aim is to test the robustness of the method compared to five-cross validation. Table 13 presents the test results for each scenarios A, B, C, D, and E. From Table 13, it can be seen that scenario B produces 94% accuracy by Gaussian SVM. The highest accuracy is also achieved by scenario E, with 96% accuracy, while scenario D produces higher accuracy than scenario C. Scenario A still produces the lowest accuracy. These results show similarity with the use of the five-cross validation technique. This test shows that the proposed method is robust against variations in the amount of training and test data.

The confusion matrix for the highest accuracy is presented in Table 14. Post-stroke MCI was successfully classified, with 100% accuracy, while post-stroke dementia and normal were classified with an accuracy of 92.3% and 94.4%, respectively. Errors occurred in the normal class detected as MCI, and the dementia class detected as MCI, but did not occur in the normal class detected as dementia. The classification simulation corroborates the significance test results that the proposed EEG characterization methods can be used to support the clinical diagnosis of early detection of post-stroke dementia and evaluation of the severity of dementia.

Performance evaluation of the proposed method on the Alzheimer’s dataset was not limited to a significance test. Evaluation using classifier techniques was also applied to determine the performance of the proposed method. Coherence and spectral entropy features were used as predictors in classification. The results were compared with similar studies using the same dataset. Details of the test results and comparison with previous studies are presented in Table 15. Table 15 shows that the proposed method produces the highest accuracy of 85.2% using cubic SVM. The comparative study shows that the proposed method outperforms the previous study by Hadiyoso et al. [37]. Meanwhile, the accuracy is slightly lower compared to the study by Kashefpoor et al. [17]. However, their study only used eighteen samples (nine normal and nine MCI). For the same sample, their study used half the length for training and the other half for testing. Meanwhile, in this proposed study, tests and training data were used from different subjects.

## 4. Discussion

In this study, EEG signal processing was carried out in post-stroke patients to characterize patients with cognitive impairment. The feature extraction method can describe brain activity changes so that EEG signals can be estimated that describe normal conditions, mild cognitive disorders, and dementia.

The power spectral characterization showed the differences in the power of the delta, alpha, and beta waves. The group with cognitive impairment showed a higher delta wave power pattern than the normal group, followed by a decrease in the power of alpha and beta waves. The average relative power of each group showed that the highest significance was found in the delta and beta rhythms. The delta power in dementia and mild vascular cognitive groups tended to be higher than in the normal group. These results confirmed the study by Meghdadi et al., that there is an increase in the delta and theta power in the elderly with dementia [39]. 

Meanwhile, the beta wave power of the normal group was higher than mild vascular cognitive impairment and dementia. In the studies by Seokbeen Lim et al. and Hendrayana et al., beta waves increased during concentration [40,41]. Significant differences with *p* < 0.05 were found in the fronto-temporo-parietal region [40]. Decreased power of beta rhythms in MCI and dementia is associated with reduced focus or concentration on working memory tasks. These findings suggest that decreased beta-band activity in low-performing patients reflects the difficulty in activation and deficits in maintaining concentration processes [42]. Jang et al.’s study showed that increasing beta power was associated with increased cognitive function [43]. The strength of the delta and beta rhythms showed a linear relationship with the severity of dementia. These results demonstrate similar characteristics to the resting EEG recordings presented in the previous section. The characteristic differences between normal subjects and patients with cognitive impairments can be caused by the degradation of neurons that affect local oscillatory activity and connectivity [44]. EEG patterns with dominant delta rhythms are found in individuals during deep sleep or in those with brain disorders [45,46]. 

Interhemispheric observations showed that the mean coherence values in patients with cognitive impairment tended to be lower than in normal subjects (CohDem < CohMCI < CohNormal) for all electrode pairs; significantly in pairs F7-F8, T3-T4, T5-T6, and P3-P4 (*p* < 0.05). These represent the temporo-parietal lobe region. Our findings confirm that the study by Al-Qazzaz et al. [23,47], which investigated signal complexity in stroke patients related to cognitive impairment, showed a significant decrease in the temporal region. We assume that stroke-associated dementia patients have a number of damaged neurons and synapses in this region. In the investigation of intrahemispheric coherence, we also found decreased coherence in patients compared to the normal control. The decrease in coherence values between brain regions is strongly correlated with cognitive impairment, as reported in previous studies [48,49,50].

The analysis of brain connectivity using coherence describes the synchronization or coordination between brain areas. Coherence analysis was performed on the interhemisphere and intrahemisphere, describing the relationship between the right and left hemispheres of the brain and the same area of the brain. Coherence in the post-stroke group with cognitive impairment tends to be lower than coherence in normal elderly patients. The most likely reason for the lower coherence is the death of many neurons and the degeneration of synapses, leading to a decrease in cortical connectivity function [51,52]. The results of the multiple comparison test for the three groups showed significant interhemispheric and intrahemispheric coherence, especially in the frontal and temporal areas. These results make coherence analysis a reliable predictor in the classification test stage.

The complexity calculation results show that post-stroke patients with cognitive impairment tend to have lower signal complexity than the normal group (SpecEn_Dem_. < SpecEn_MCI_ < SpecEn_Normal_) and (SpecDE_Dem._ < SpecDE_MCI_ < SpecDE_Normal_). Another issue of observing SpecEn and SpecDE values is that there is an association between decreased signal complexity and dementia severity, as reported in the study of Al-Qazzaz et al. This finding confirms the results of previous studies, that a worsening of dementia will be followed by a decrease in signal complexity. The results of the analysis of memory-related brain activity recordings showed similar network dynamics [53], as evidenced by the consistency of SpecEn and SpecDE values. SpecEn and SpecDE results show a change in the power spectral frequency distribution. This is associated with a slowing of the EEG of MCI and dementia patients [54,55]. The most likely physiological interpretation to explain this is the occurrence of significant brain cholinergic deficits as the basis for symptoms of cognitive decline. Cholinergics regulate spontaneous activity at low frequencies followed by loss of neurotransmitters, leading to a slowing of nerve oscillations. The results of the significance test also showed significant differences between groups, especially SpecDE, which resulted in better discrimination features than the other two methods. Signal complexity characterization can be a supporting criterion in the classification test stage.

Quantitative EEG (QEEG) can be an essential tool to simplify the analysis of digital EEG tools. QEEG, in this study, uses power spectral, coherence, and complexity analysis. The quantification results show the characteristics of discrimination between normal, post-stroke mild cognitive impairment, and post-stroke dementia. Spectral analysis, coherence, and complexity can describe the condition of the brain with decreased cognitive function. From the proposed characterization method, it can be estimated whether there are brain abnormalities related to cognitive function. Furthermore, with a combination of EEG characterization methods, the severity of dementia can be classified as a diagnostic support tool in the early detection of post-stroke vascular dementia. Future research can perform feature selection of coherence and spectral dispersion entropy to obtain essential features to reduce the number of features, while still producing optimum classification accuracy.

## 5. Conclusions

This study developed a quantitative EEG (QEEG) method to characterize EEG waves in post-stroke patients at risk of developing vascular dementia. QEEG methods used for analysis included spectral power, coherence, and signal complexity. These methods were used to improve the function of a digital EEG device that described the brain’s functionality for early identification of cognitive impairment due to vascular disease that leads to cerebral blood vessels.

In developing the method, this study involved three test groups: normal subjects, post-stroke patients with mild cognitive impairment (MCI), and post-stroke dementia patients. The subject criteria used in this study were based on recommendations. They were selected by a neurobehavior consultant neurologist after clinical, neuropsychological, and brain imaging examinations were carried out. The recommendations for normal and impaired cognition were based on neuropsychological examination by a neurologist using the MoCA assessment. Clinical examination, psychology, and EEG recordings were conducted at Hasan Sadikin Hospital, Bandung. This research received ethical approval from the hospital ethics committee; number LB.02.01/X.6.5/272/2019.

Power spectral characterization showed that patients with cognitive impairment had higher delta relative power and decreased alpha and beta relative power than the normal group. The most significant differences in delta and beta waves were found at the frontal, temporal, and parietal electrodes (*p*-value < 0.05). This characterization also demonstrated an association between EEG signal strength and dementia severity.

Another analysis was the interhemispheric and intrahemispheric coherences, which describe the connectivity of brain tissue. Observations of interhemispheric coherence showed that the mean coherence value in patients with cognitive impairment was lower than in normal subjects (CohDem < CohMCI < CohNormal). Significance (*p* < 0.05) was found in the frontal-temporo-parietal lobe electrode pair. In the investigation of intrahemispheric coherence, a decrease in coherence was found in patients compared to normal subjects. Significant differences existed in the local and distal intrahemispheric coherence electrode pairs, including frontal, central, and temporal. These results represent the consistency of interhemispheric coherence measurements, where the central and temporal regions experience decreased coherence due to the failure of functional connectivity. Thus, the decrease in coherence values between brain regions strongly correlates with disorders related to cognitive function.

Meanwhile, the SpecEn and SpecDE analyses showed that the post-stroke patient group with impaired cognition tended to produce a lower signal entropy than the normal group. Physically, the patient group had more regular EEG signals than the normal group. The multiple comparison tests showed that the SpecDE analysis provides discriminatory significance for the case of three groups that are superior to SpecEn. It was indicated by a *p*-value <0.05 in normal cases vs. post-stroke MCI, and post-stroke MCI vs. post-stroke dementia was more commonly observed.

Characteristic differences between normal conditions and patients with impaired cognition may be due to different brain conditions due to neuronal degradation. Delta waves with dominant strength occur when the state of deep sleep or the conscious state of someone with a brain disorder. The explanation for the lower coherence in this group of patients is the death of large numbers of neurons and the degeneration of synapses, leading to a decrease in cortical connectivity function. It can underlie disturbances in functional interaction or coordination between brain regions.

At the classification stage to test the proposed method, prediction simulations were carried out using SVM and k-NN. This study succeeded in classifying normal EEG, mild cognitive impairment, and dementia, with the highest accuracy of 96%. The highest accuracy was achieved using Gaussian SVM by combining coherence and SpecDE features. Cases classified as normal and dementia could be perfectly classified. Meanwhile, for the classification of normal vs. post-stroke MCI and post-stroke MCI vs. post-stroke dementia, the accuracy of each was 94.4%. From the QEEG method developed, the EEG tool could be used to evaluate vascular dementia in post-stroke patients. This study could support clinical diagnosis in the early detection and evaluation of the severity of vascular dementia in post-stroke patients. In future research, it is recommended to collect data on a larger population so that the reliability of the proposed method can be analyzed.

## Figures and Tables

**Table 1:** Clinical data of research subjects.

Index	Normal	Post Stroke-MCI	Post Stroke-Dementia
Number of samples	18	19	13
Gender (M/F)	10/8	9/10	8/5
Age (std. dev.)	57.18 ± 4.9	58.84 ± 5.62	59.7 ± 5.76
Education (years)	13.45 ± 3.9	11.68 ± 4.02	12.38 ± 4.4
MoCA-INA score	26.5 ± 1.33	22.16 ± 2.22	12.23 ± 3.77
Hypertension	N	Y	Y
Diabetes	N	Y	Y

**Table 2:** Wavelet sub-band and corresponding EEG frequency.

Level	Sub-Band	Frequency Band (Hz)	EEG Frequency	Amplitude [28]
2	D2	30–60 Hz	Gamma (γ)	<50 µV
3	D3	15–30 Hz	Beta (β)	<30 µV
4	D4	7.5–15 Hz	Alpha (α)	<50 µV
5	D5	3.75–7.5 Hz	Theta (θ)	0–20 µV
5	A5	0–3.75 Hz	Delta (δ)	0–100 µV

**Table 3:** Interhemispheric and intrahemispheric coherence electrode pairs.

Interhemispheric	Left-Intrahemispheric		Right-Intrahemispheric	
Fp1-Fp2	Fp1-F3	F7-T3	Fp2-F4	F8-T4
F3-F4	Fp1-F7	F7-P3	Fp2-F8	F8-P4
F7-F8	Fp1-C3	F7-T5	Fp2-C4	F8-T6
C3-C4	Fp1-T3	F7-O1	Fp2-T4	F8-O2
T3-T4	Fp1-P3	C3-T3	Fp2-P4	C4-T4
P3-P4	Fp1-T5	C3-P3	Fp2-T6	C4-P4
T5-T6	Fp1-O1	C3-T5	Fp2-O2	C4-T6
O1-O2	F3-F7	C3-O1	F4-F8	C4-O2
	F3-C3	T3-P3	F4-C4	T4-P4
	F3-T3	T3-T5	F4-T4	T4-T6
	F3-P3	T3-O1	F4-P4	T4-O2
	F3-T5	P3-T5	F4-T6	P4-T6
	F3-O1	P3-O1	F4-O2	P4-O2
	F7-C3	T5-O1	F8-C4	T6-O2

**Table 4:** Relative power significance test results.

Channel	RPdelta	RPtheta	RPalpha	RPbeta
FP1	0.1936	0.9075	0.3107	0.018 *
F7	0.3455	0.5639	0.4416	0.0188 *
T3	0.0036 *	0.1673	0.3103	*p* < 0.001 *
T5	0.0593	0.5221	0.3696	0.003 *
FP2	0.1294	0.8983	0.3414	0.0077 *
F8	0.2869	0.4174	0.843	0.0451 *
T4	0.0026 *	0.0593	0.9132	0.0109 *
T6	0.1192	0.487	0.7131	0.0023 *
F3	0.242	0.2704	0.6224	0.0875
C3	0.2364	0.3268	0.9923	0.0945
P3	0.2609	0.2752	0.8701	0.0218 *
O1	0.6664	0.595	0.9407	0.013 *
F4	0.0449 *	0.6309	0.7393	0.0109 *
C4	0.1035	0.283	0.7979	0.1104
P4	0.0255 *	0.3434	0.4106	0.0017 *
O2	0.0222 *	0.5785	0.7762	0.006 *
FZ	0.429	0.6029	0.398	0.0161 *
CZ	0.8874	0.6813	0.5812	0.0047 *
PZ	0.3649	0.1791	0.4334	*p* < 0.001 *

* *p*-value < 0.05.

**Table 5:** The value of interhemispheric coherence with *p* < 0.05.

Electrode Pairs	*p*-Value
F7-F8	*p* < 0.001
T3-T4	*p* < 0.001
T5-T6	*p* < 0.001
P3-P4	0.0011
O1-O2	*p* < 0.001

**Table 6:** The right intrahemispheric coherence value resulted in *p* < 0.05.

Pair	*p*-Value	Pair	*p*-Value
Fp2-C4	0.074466	F8-P4	0.008868
Fp2-T4	0.001599	F8-T6	0.004805
Fp2-P4	0.000061	F8-O2	0.000113
Fp2-T6	0.011516	C4-T6	0.009819
Fp2-O2	0.003125	C4-O2	0.001699
F4-T4	0.004631	T4-P4	0.008749
F4-T6	0.006452	T4-T6	0.013341
F4-O2	0.002523	T4-O2	0.000052
F8-C4	0.028184	P4-T6	0.000409
F8-T4	0.027075	T6-O2	0.005392

**Table 7:** The left intrahemispheric coherence value resulted in *p* < 0.05.

Pair	*p*-Value	Pair	*p*-Value
Fp1-F3	0.000877	F7-P3	0.00169
Fp1-F7	0.000349	F7-T5	0.015812
Fp1-C3	0.003417	F7-O1	0.021341
Fp1-T3	0.000000	C3-O1	0.000104
Fp1-P3	0.013351	T3-P3	0.01515
Fp1-T5	0.000568	T3-O1	0.00085
Fp1-O1	0.038215	P3-T5	0.004883
F3-F7	0.033907	P3-O1	0.003348
F3-T3	0.011276	T5-O1	0.038446
F3-P3	0.000494		
F3-T5	0.007562		
F3-O1	0.000019		

**Table 8:** SpecEn and SpecDE significance test results for each EEG channel.

Electrode	*p*-Value	
	SpecEn	SpecDE
Fp1	0.0032	0.0001
F7	0.0000	0.0015
T3	0.0004	0.0004
T5	0.0039	0.0000
Fp2	0.0033	0.0006
F8	0.0003	0.0001
T4	0.0002	0.0019
T6	0.0002	0.0000
F3	0.0001	0.0093
C3	0.0007	0.0000
P3	0.0084	0.0000
O1	0.0298	0.0000
F4	0.0001	0.0227
C4	0.0001	0.0000
P4	0.0012	0.0000
O2	0.0024	0.0006
Fz	0.0003	0.0004
Cz	0.0035	0.0034
Pz	0.0226	0.0029

**Table 9:** The results of post hoc multiple comparison (SpecEn).

	(95% Confidence Level)									
Comparison	Fp1	F7	T3	T5	Fp2	F8	T4	T6	F3	C3
Normal vs. Stroke-MCI	0.141	0.047 *	0.207	0.108	0.247	0.071	0.144	0.030 *	0.079	0.173
Normal vs. Stroke-Dementia	0.002 *	0.000 *	0.000 *	0.003 *	0.002 *	0.000 *	0.000 *	0.000 *	0.000 *	0.000 *
Stroke-MCI vs. Stroke-Dementia	0.174	0.020 *	0.026 *	0.251	0.097	0.062	0.019 *	0.11	0.020 *	0.044 *
	P3	O1	F4	C4	P4	O2	Fz	Cz	Pz	
Normal vs. Stroke-MCI	0.207	0.262	0.042 *	0.19	0.148	0.213	0.011 *	0.020 *	0.17	
Normal vs. Stroke-Dementia	0.006 *	0.024 *	0.000 *	0.000 *	0.001 *	0.002 *	0.000 *	0.004 *	0.020 *	
Stroke-MCI vs. Stroke-Dementia	0.232	0.395	0.054	0.011 *	0.071	0.09	0.315	0.154	0.513	

* *p*-value < 0.05.

**Table 10:** The results of post hoc multiple comparison (SpecDE).

	Significant (95% Confidence Level)									
Comparison	Fp1	F7	T3	T5	Fp2	F8	T4	T6	F3	C3
Normal vs. Stroke-MCI	0.017 *	0.058 *	0.020 *	0.084	0.334	0.152	0.784	0.102	0.368	0.000 *
Normal vs. Stroke-Dementia	0.000 *	0.000 *	0.000 *	0.000 *	0.000 *	0.000 *	0.000 *	0.000 *	0.003 *	0.000 *
Stroke-MCI vs. Stroke-Dementia	0.023 *	0.118	0.079	0.000 *	0.006 *	0.008 *	0.000 *	0.011 *	0.080	0.148
	P3	O1	F4	C4	P4	O2	Fz	Cz	Pz	
Normal vs. Stroke-MCI	0.014 *	0.004 *	0.337	0.017 *	0.028 *	0.148	0.083	0.045 *	0.112	
Normal vs. Stroke-Dementia	0.000 *	0.000 *	0.007 *	0.000 *	0.000 *	0.000 *	0.000 *	0.003 *	0.000 *	
Stroke-MCI vs. Stroke-Dementia	0.000 *	0.002 *	0.159	0.019 *	0.011 *	0.031 *	0.021 *	0.436	0.071	

* *p*-value < 0.05.

**Table 11:** Classification test scenario.

Scenario	Classification Feature (Predictor)	Attributes
A	Power spectral	Relative power all bands (19 each)
B	Coherence	Inter- and intrahemispheric (right, left)
C	Complexity SpecEn	Spectral entropy all channels
D	Complexity SpecDE	Spectral dispersion entropy all channels
E	Coherence, complexity SpecDE	Feature Scenario B + D

**Table 12:** Classification results for each scenario.

Scenario	Classifier	Accuracy	Specificity	Sensitivity	Precision	F1-Score
A	Linear SVM	50	73.7	48	56	48.9
	Quadratic SVM	52	75.3	52.3	53	52.6
	Cubic SVM	50	74.9	51.1	50.1	50.2
	Gaussian SVM	42	69.3	39.3	49.9	38.7
	fine k-NN	30	65.1	31	30.4	30.2
	medium k-NN	46	73.9	49.9	49.7	45.4
	cubic k-NN	48	74.7	51.7	53.4	47.9
B	Linear SVM	78	88.2	73.9	87.8	74.7
	Quadratic SVM	80	89.3	75.8	87.3	76
	Cubic SVM	80	89.3	75.1	87.4	74.1
	Gaussian SVM	94	96.8	93.7	95.5	94.3
	fine k-NN	70	83.1	63.7	77.2	61.6
	medium k-NN	70	83.9	63.7	82.3	58.4
	cubic k-NN	68	82.9	61.2	NAN	NAN
C	Linear SVM	46	72.2	44.8	45.3	44.4
	Quadratic SVM	44	71.4	43.6	44.6	44.1
	Cubic SVM	36	67.1	34.2	35.4	34.3
	Gaussian SVM	48	73.3	46.5	47.7	46.8
	fine k-NN	36	67.8	35.7	35.8	35.7
	medium k-NN	54	75.9	51.1	56.3	50.6
	cubic k-NN	50	74.4	49.2	49.1	49
D	Linear SVM	78	88.4	75.7	81	76.6
	Quadratic SVM	76	87.4	74.6	79.2	75.8
	Cubic SVM	66	82.4	65	66.7	65.4
	Gaussian SVM	76	87.3	73.1	79.5	73.9
	fine k-NN	60	79.9	58.1	57.4	57.7
	medium k-NN	80	89.3	77.3	86.8	78.8
	cubic k-NN	74	86.4	71.2	76.7	72.2
E	Linear SVM	80	89.3	75.8	88.5	76.3
	Quadratic SVM	84	91.4	80.9	90.1	82.2
	Cubic SVM	84	91.4	80.9	90.1	82.2
	Gaussian SVM	**96**	**97.9**	**95.6**	**96.8**	**96.1**
	fine k-NN	74	85	67.7	79.2	66.7
	medium k-NN	74	86	68.1	86.5	65.1
	cubic k-NN	72	85.2	68.1	79.7	67.1

**Table 13:** Accuracy for each scenario using ten-cross validation.

Classifier	Accuracy (%) Each Scenario				
	A	B	C	D	E
Linear SVM	50	82	48	76	84
Quadratic SVM	54	78	48	70	80
Cubic SVM	52	78	32	66	80
Gaussian SVM	42	**94**	52	76	**96**
fine k-NN	38	70	44	56	72
medium k-NN	48	66	42	76	70
cubic k-NN	38	66	48	72	76

**Table 14:** The confusion matrix of the highest accuracy.

		Predicted		
		Normal	Stroke-MCI	Stroke-Dementia
Actual	Normal	17	1	0
	Stroke-MCI	0	19	0
	Stroke-Dementia	0	1	12

**Table 15:** Accuracy for AD dataset and comparison with previous studies.

Study	Feature Extraction Method	Classifier	Number of Sample Test	Accuracy (%)
Kashefpoor et al. [17]	Spectral Analysis	Neurofuzzy(NF)-k-NN	18 (9 normal, 9 MCI)	88.8
Hadiyoso et al. [37]	Spectral Analysis	k-NN	27 (16 normal, 11 MCI)	81.5
Proposed	Coherence + Complexity	Linear SVM	27 (16 normal, 11 MCI)	77.8
		Quadratic SVM		77.8
		Cubic SVM		85.2
		Gaussian SVM		81.5
		Fine k-NN		77.8
		Medium k-NN		66.7
		Cubic k-NN		81.5

## Data Availability

Since this research involves human subjects and is related to ethical approval, data availability is based on request to the corresponding author.
